# Pulmonary Fibrosis among World Trade Center Responders: Results from the WTC Health Registry Cohort

**DOI:** 10.3390/ijerph16050825

**Published:** 2019-03-07

**Authors:** Jiehui Li, James E. Cone, Robert M. Brackbill, Ingrid Giesinger, Janette Yung, Mark R. Farfel

**Affiliations:** New York City Department of Health and Mental Hygiene, World Trade Center Health Registry, New York City, NY 10013, USA; jcone@health.nyc.gov (J.E.C.); rbrackbi@health.nyc.gov (R.M.B.); igiesinger@health.nyc.gov (I.G.); jyung@health.nyc.gov (J.Y.); mfarfel@health.nyc.gov (M.R.F.)

**Keywords:** World Trade Center disaster, pulmonary fibrosis, dust

## Abstract

Dust created by the collapse of the World Trade Center (WTC) towers on 9/11 included metals and toxicants that have been linked to an increased risk of pulmonary fibrosis (PF) in the literature. Little has been reported on PF among WTC responders. This report used self-reported physician diagnosis of PF with an unknown sub-type to explore the association between levels of WTC dust exposure and PF. We included 19,300 WTC responders, enrolled in the WTC Health Registry in 2003–2004, who were followed for 11 years from 2004 to 2015. Exposure was defined primarily by intensity and duration of exposure to WTC dust/debris and work on the debris pile. Stratified Cox regression was used to assess the association. We observed 73 self-reported physician-diagnosed PF cases, with a PF incidence rate of 36.7/100,000 person-years. The adjusted hazard ratio (AHR) of PF was higher in those with a medium (AHR = 2.5, 95% CI = 1.1–5.8) and very high level of exposure (AHR = 4.5, 95% CI = 2.0–10.4), compared to those with low exposure. A test for exposure—response trend was statistically significant (*P*_trend_ = 0.004). Future research on WTC dust exposure and PF would benefit from using data from multiple WTC Health Program responder cohorts for increased statistical power and clinically confirmed cases.

## 1. Introduction

The collapse of the World Trade Center (WTC) towers resulting from the terrorist attack on 11 September 2001 (9/11) produced a dense cloud of dust and debris that spread widely and contained toxic substances including heavy metals (e.g., titanium), silica, asbestos fibers, and wood dust [1,2]. WTC responders were potentially highly exposed as a result of performing rescue and recovery work on the WTC site including searching for remains, firefighting and debris removal work that continued through June 2002 [3]. Of those involved in the rescue/recovery activities, nearly half were directly exposed to the dust cloud (blackout conditions) on 9/11 [3]. In total, 93% of WTC responders had commenced work on the site by the end of September, 2001 [3].

Short and longer-term health effects of 9/11 associated with intense dust/fume exposure include a number of respiratory diseases or symptoms, such as asthma, chronic obstructive pulmonary disease (COPD) and upper or lower respiratory symptoms [4,5,6,7,8,9]. A small number of cases of pulmonary fibrosis (PF) among WTC responders were reported in the first eight years following 9/11 [4,10,11]. PF is an interstitial lung disease, characterized by scarring (fibrosis) formed over time in the structural lung tissue between alveoli (or air sacs) [12]. Lung biopsies in four out of seven previously healthy WTC responders in a case series study revealed histologic patterns that were consistent with interstitial disease, following imaging that identified suspected interstitial disease after 9/11 exposure [10]. In another case series from the WTC Environmental Health Center (WTC EHC), five of six patients, who underwent surgical biopsy based on CT findings suggestive of diffuse interstitial disease of fibrosis, had pathologic findings of mild to moderate patchy interstitial fibrosis [11]. Moreover, these two case series that included mineralogical analyses of the available tissues found the presence of metals, silica, aluminum silicates, carbon nanotubes, chrysotile asbestos and calcium phosphate or sulfate [10,11]. Four cases of bilateral pulmonary fibrosis were also reported among New York City firefighters [4]. All of these initial observations from individual patients provided important clues to the occurrence of this rare disease among 9/11-exposed individuals.

WTC dust may be responsible for the reported PF cases as the substances known to be associated with PF in occupational studies include heavy metals (e.g., titanium), silica, and wood dust [13,14,15,16,17,18,19], which were also found in WTC dust [1,2]. However, little is known about the occurrence of PF in this population over time and whether the level of WTC dust exposure would be associated with PF in an observational study in which WTC responders with PF could be compared to those who do not have PF.

Using self-reported survey data on PF from the World Trade Center Health Registry [20], we investigated the incidence of PF and assessed the association between WTC dust exposure and PF in a large longitudinal cohort of WTC responders during an 11-year median follow-up period. We hypothesized that the intensity of WTC dust exposure is associated with increased risk of PF.

## 2. Materials and Methods

### 2.1. Study Population

The World Trade Center Health Registry (Registry) was established in 2003–2004 as an exposure cohort study designed to monitor the long-term health effects of the September 11 attacks among rescue/recovery workers and persons who lived, worked, or attended school in lower Manhattan. The Registry’s methods have been published elsewhere [5,20]. Briefly, in 2003–2004 (enrollment, Wave 1 survey) over 71,000 people were enrolled and completed a telephone (95%) or in-person (5%) interview. Participants were identified either through lists provided by employers, government agencies, and other entities or by responding to an outreach campaign (30% list identified, 70% self-identified). At enrollment, data on demographics, exposure incurred during and after 9/11, and health information were collected. Since Wave 1 (W1), three follow-up surveys have been conducted, in 2006–2007 (Wave 2, W2), 2011–2012 (Wave 3, W3), and 2015–2016 (Wave 4, W4).

Physician diagnosis of PF was first inquired about in W3 and repeated in W4; therefore, only those who participated in W3 or W4 and completed a question related to PF diagnosis were included in this study. To test our hypothesis, we focused our study sample on WTC responders, who presumably experienced more intense dust exposure than those not involved with rescue/recovery. We defined WTC responders in this present study as those who worked at least one shift at the WTC site providing rescue, recovery, clean-up, construction, or support services from 11 September 2001 to 30 June 2002, and who provided information related to work and exposure at the WTC site anytime during the 9 months of rescue/recovery operations. Those younger than 18 years on 11 September 2001 or of unknown age were excluded. The final sample of responders included firefighters, police, emergency medical services/medical/disaster personnel, construction/engineering workers, affiliated or unaffiliated volunteers, other government workers, and sanitation workers.

The Centers for Disease Control and Prevention (CDC) and the New York City Department of Health and Mental Hygiene institutional review boards approved the World Trade Center Health Registry protocol.

### 2.2. PF Definition

Self-reported physician-diagnosed PF between 2004 and 2015 was the endpoint of interest. In W3 and W4, we inquired about whether the enrollee had ever been told by a physician that they had PF and the associated year of diagnosis. If WTC responders participated in both W3 and W4, the first report of the year of diagnosis was used, and PF was defined as consistent PF information being provided on both surveys. If responders participated in W3 or W4 but not both, the year of diagnosis reported at the respective survey was used. As we only had the year of diagnosis available, the midpoint of the reported year (June 30) was used as the diagnosis date. To reduce the subjectivity of self-reported physician-diagnosed PF, we added self-reported shortness of breath in the 30 days prior to reporting PF to the case definition. Shortness of breath is one of the most common symptoms presented by PF patients, due to scarring and thickening in the lung tissue. We excluded self-reported PF cases diagnosed prior to Registry enrollment or 2004 and those participants who had no symptoms of shortness of breath in the 30 days prior to reporting PF, had missing years of diagnosis, or inconsistent information on PF diagnosis. Individuals with sarcoidosis were excluded because not all cases of sarcoidosis are pulmonary and we were unable to differentiate these cases within our study. These included sarcoidosis identified either from the Registry’s in-depth study through a medical record review [21], or from the New York State Department of Health’s SPARCS (Statewide Planning and Research Cooperative System) inpatient data (2000–2015) using ICD-9 diagnosis code 135 or ICD-10 D86. Only 20% of persons with sarcoidosis develop fibrotic lung disease [22], although there has been a demonstrated association between WTC exposure and sarcoidosis among responders [21,23,24].

### 2.3. WTC Dust Exposure and Covariates

Physical exposure to dust at the WTC site was based on self-reported WTC exposures that were collected at enrollment [25]. The WTC dust exposure composite score, developed using a modified Delphi method, took information on date of arrival, duration of work at the site, dates or time period working on the pile and being near the WTC site, or exposure to dust/debris resulting from the collapse of buildings on 9/11 into account. The sum of WTC dust exposure scores for each WTC responder was analyzed both as a continuous variable (actual sum scores) for trend and as an ordinal scale variable for analysis of PF risk at each exposure quartile. The latter, based on quartiles of the summed points, was categorized as: Low (Q1 ≤ 25%), medium (Q2), high (Q3), and very high (Q4 > 75%).

Covariates identified in the literature as potential risk factors were included in the multivariable analysis, including age, gender, race and ethnicity, income, and smoking status (current, former or never), which were reported at enrollment. We also examined the prevalence of comorbidities in our WTC responders by PF status, as some physical and mental health conditions frequently co-exist or are highly prevalent in persons with idiopathic PF which is the most common type of PF [26]. Medical conditions were based on self-reports in W3 or W4 of having been told by a medical professional that the participant had the condition. Conditions included chronic obstructive pulmonary disease (COPD), diabetes, gastroesophageal reflux disease (GERD), obstructive sleep apnea (OSA), asthma, depression, anxiety, or post-traumatic stress disorder (PTSD).

### 2.4. Statistical Analysis

Incident rate of PF by characteristics and WTC exposure were summarized using crude incidence rates and expressed as PF per 100,000 person-years of follow-up. The association of WTC dust exposure with time to PF diagnosis was assessed using Cox proportional hazards regression modeling. To test for proportionality, we assessed time dependent covariates by creating interactions of all predictors and a function of survival time and included these in the model. We found that “smoking” did not satisfy the proportional hazard assumption, and therefore, carried out a stratified Cox proportional hazards regression for the analysis. In the stratified Cox model, “smoking” was controlled by stratification [27]. Results of multivariable analysis were expressed as adjusted hazard ratios (AHR) with 95% confidence intervals (CI). Person-years of follow-up began on the date of enrollment and ended at the time of PF diagnosis or were censored on the last follow-up (W3 or W4 survey completion date, whichever was most recent). All statistical analyses were performed using SAS software (SAS Institute, Cary, NC, USA V9.4). Two-sided *p* values of less than 0.05 were considered significant.

## 3. Results

Of a total of 29,721 WTC responders, enrolled in the Registry in 2003–2004, 19,300 WTC responders met eligibility criteria for data analyses. Excluded were those younger than 18 years old on 9/11 or deceased prior to enrollment (*N* = 205), non-participants of W3 and W4 (*N* = 8181), those with missing reports of PF or year of diagnosis (*N* = 1172), those with inconsistent reports, missing information on shortness of breath or pre-2004 PF (*N* = 837), sarcoidosis (*N* = 1), or missing data on WTC exposure (*N* = 25).

The characteristics of the study sample at enrollment are presented in Table 1. The median age of responders at enrollment was 41 years (interquartile range, 34–48 years). Responders included in this study were predominantly male (77.8%), non-Latino white (75.6%), and had an annual household income ≥$50,000 (70.2%). With respect to smoking status at baseline, the percent of former smokers was 29.3% among non-Latino whites, 19.9% in non-Latino blacks, 24.6% in Latinos, and 16.4% in Asians; the percent of current smokers was 15.2% among non-Latino whites, 15.7% in non-Latino blacks, 17.7% in Latinos, and 15.3% in Asians.

A total of 73 incident cases of PF were reported among 19,300 responders over the study period between 2004 and 2015, which totaled 199,113.6 person-years. The overall incidence rate of PF was 36.7 per 100,000 person-years. The PF incidence rate was higher in the older age groups, males, Latino or Asian, those with low household income, current smokers and those with higher WTC exposure (Table 2).

In multivariable analysis, taking socio-demographics into account, we observed an exposure—response relationship between WTC dust exposure and PF (Table 3) (*p* for linear trend = 0.004). Compared to those with a low level (Q1) of exposure, approximately 2.5 times as many WTC responders in the 2nd and 3rd quantiles were experiencing PF, and those in Q4 (the highest level of exposure) experienced the highest AHR during the follow-up period. AHR at each quartile was significantly elevated compared to that at the lowest level.

Distribution of comorbidity by self-reported physician-diagnosed PF is presented in Table 4. The prevalence of co-existing physical conditions (e.g., GERD, OSA, asthma, COPD or emphysema, and diabetes) and mental disorders (e.g., depression, PTSD and anxiety) among PF patients was significantly higher than it was in those without PF (*p* = 0.030 for diabetes, and *p* < 0.001 for all other conditions). Of these comorbid conditions, GERD (69.4%) and OSA (68.5%) had the highest prevalence among individuals with PF.

## 4. Discussion

In this longitudinal WTC exposure cohort of 19,300 responders, a total incidence rate of PF was 36.7 per 100,000 person-years between 2004 and 2015. Although little is reported on prevalence of PF in general populations, we do know that idiopathic pulmonary fibrosis (IPF), the most common type of PF, has an estimated prevalence of 14–63 cases per 100,000 population, and incidence of 6.8–17.4 per 100,000 population in the United States, dependent on the case definition [28]. Supporting our hypothesis, we observed that WTC dust exposure increased the risk of PF in an exposure—response relationship among WTC responders. AHR at each quartile was significantly elevated compared to that at the lowest quartile.

### 4.1. WTC Dust and PF

WTC dust may be a plausible source of PF risk due to its unique characteristics and components that are known to be linked to PF. Particle size (>90% between 2.5 and 100 µm) of WTC dust can be inhaled and deposited in the airways and lungs when the airborne concentrations are high [1,29]. Second, the highly alkaline nature of the pulverized building materials can cause both physical and chemical irritation to the respiratory and gastroesophageal epithelia [1,30]. Third, readily airborne resuspension can increase surface re-contamination levels of WTC dust both indoors and outdoors [30]. Of the 287 chemicals and chemical groups identified from environmental sampling of the area around the WTC in New York City [31], asbestos and glass fibers, crystalline silica, various metals, volatile organic compounds, polychlorinated polycyclic compounds, and polycyclic aromatic hydrocarbons were included. Some of the chemical toxic components identified from WTC dust, such as heavy metals (e.g., titanium), silica, asbestos fibers, and wood dust, have been linked to PF [2,32]. Taken together with early reports of PF among WTC exposed patients [10,11], the unique characteristics of WTC dust and its toxic components might explain our observed significant exposure—response effect of WTC dust exposure on PF among WTC responders.

Recently, a review of inflammatory response to inhalation of causative chemicals in WTC was suggested as a possible mechanism of a link between WTC dust and PF [32]. A dysregulated inflammatory response can gradually evolve into a pathogenic fibrotic response when inflammation becomes persistent [33]. Our findings of the association between WTC dust exposure and PF remain to be confirmed by an in-depth study using clinically confirmed cases over an extended follow-up period.

### 4.2. Comorbidity in PF

WTC responders with PF had significantly higher pulmonary and non-pulmonary comorbidities compared with those without PF, aligning with the high prevalence of co-existing physical or mental health conditions in idiopathic PF (IPF) patients in the literature. Raghu et al., in his review, found that prevalence of COPD in IPF patients ranged from 6 to 67%, of obstructive sleep apnea from 6 to 91%, of GERD from 0 to 94%, and of diabetes from 10 to 33% [26]. Depression and anxiety are also reported to be associated with dyspnea and other measures of fibrosis severity [34]; 50% of PF patients have depression and 30% have anxiety [34]. Consistent with the literature, PF in this study frequently co-existed with the physical or mental health conditions mentioned above. The comorbidities reported in responders with PF have also been reported to be significantly associated with WTC exposure among affected populations either in WTC rescue/recovery workers or local community members [7,30,35,36]. Although 9/11-exposed populations are at high risk for comorbidities [6,9,37], the much higher prevalence of comorbidities among PF cases than in those without PF further supports the association between WTC dust and PF observed in this study and warrants further study. Additionally, the significantly high comorbidity rate among PF patients may have an important clinical impact [26,38].

### 4.3. Possible Limitations with Self-Reported PF

Our findings should be interpreted with caution. The nonspecific self-reported physician-diagnosed PF is a fundamental limitation of this study. Lack of clinical information for case confirmation, such as high-resolution computed tomography (HRCT) findings, lung function and serologic tests, raises a concern with regard to the accuracy of PF ascertainment. To help mitigate the limitation, we defined PF by adding a concurrent symptom of shortness of breath. However, we do not have data on the severity of shortness of breath in the Registry cases, as this symptom is generally progressive in PF patients. We were also unable to identify types of PF due to lack of clinical information; there are over 200 types of PF [12]. Future study using clinical diagnosis of PF is needed to corroborate our findings.

In this study we observed similar socio-demographic differences that have been reported in the prevalence of PF, which provides some confidence in the use of self-reported PF. Females, non-Latino blacks and non-smokers tend to have a lower prevalence of PF than those who are male, of a non-African origin, and smokers [28,39]. Consistent with the literature, we observed that the PF incidence rate was lower among non-Latino blacks, and much higher among Latino compared to non-Latino whites [40]. However, the higher risk for PF among Asians in this study, compared to non-Latino whites, was not expected and not consistent with findings from other studies [41]. PF among Latino and Asians was significantly associated with WTC dust exposure, independent of smoking status and other demographics at baseline. Even though smoking is a known risk factor for PF and varied by race/ethnicity in this study, smoking did not explain our observed association between WTC dust exposure and PF. Further study is warranted to explain some inconsistent findings in racial differences in PF.

### 4.4. Other Potential Biases

Potential attrition bias due to non-response to W3 and W4 may have influenced the observed results. A previous Registry study by Yu found that compared to W3 participants, non-participants were younger, more likely to be male, non-white, and to have low household income [42]. The difference in demographics between participants and non-participants is a concern. However, the non-response to W3 was not associated with WTC exposures (e.g., injury as a result of the 9/11 attacks, witnessing of traumatic events and being caught in the dust cloud on 9/11 morning), or chronic diseases reported at enrollment (e.g., asthma, emphysema, diabetes and heart disease) [42]. Thus, attrition bias in our study may be minimal. In addition, knowing that early treatment is crucial to improve PF prognosis [43], participation in W3 or W4 might have been partly affected by the survival and severity of the PF condition, which we were not able to assess due to lack of information on PF treatment.

Some of the WTC responders enrolled in the Registry may also have enrolled in the WTC Health Program for medical monitoring and treatment. The availability of the WTC Health Program that provides health monitoring through periodic visits to responders and includes a chest radiograph [42,44] may have provided increased screening for lung disease to WTC responders compared to screening provided to those not exposed or the general public, and thus may lead to potential detection bias. However, such bias would not be expected to differ between those with a lower exposure level and those with a higher level of exposure, as all WTC responders meet the WTC Health Program criteria for monitoring.

The development of PF is considered to be multifactorial [45]; environmental or occupational exposure prior to, or outside of, 9/11 rescue recovery efforts may have contributed to disease development and progression. Lack of control for potential confounders among responders such as other occupational exposure, family history, history of viral infections [46] or genetic factors might bias the observed results. However, data on these potential confounders were not available.

## 5. Conclusions

Despite the limitations, this study found evidence of the association between WTC dust and PF risk among WTC responders, adding evidence to the literature of occupational exposure and self-reported PF. Specifically, PF may be another 9/11-related health condition among the exposed population. PF is an uncommon but serious condition that often co-exists with other health conditions that the 9/11-exposed population is at risk of developing. Awareness and early detection of PF among at-risk 9/11-exposed populations may slow the progress of the condition by early intervention. While on-going surveillance of PF is needed, in-depth research using medically-verified cases and pooled cases from multiple WTC Health Program cohorts (to increase sample size) would help to better understand the relationship between WTC dust exposure and PF.

## Figures and Tables

**Table 1:** Characteristics and World Trade Center (WTC) dust exposure of study sample at enrollment.

Variable at Enrollment	No.	%
Total	19,300	100
Age, year		
<45	10,564	54.7
45–64	8205	42.5
≥65	531	2.8
Gender		
Male	15,020	77.8
Female	4280	22.2
Race/Ethnicity		
Non-Latino white	14,586	75.6
Non-Latino black	1380	7.2
Latino	2265	11.7
Asian	426	2.2
Other/unknown	643	3.3
Household income ($)		
<50,000	4261	22.1
≥50,000	13,549	70.2
Missing	1490	7.7
Smoking status		
Never	10,849	56.2
Former	5332	27.6
Current	2996	15.5
Missing	123	0.6
WTC dust/debris exposure level		
Quantile 1 (Low)	4772	24.7
Quantile 2 (Medium)	4670	24.2
Quantile 3 (High)	4800	24.9
Quantile 4 (Very high)	5058	26.2

**Table 2:** Incidence rate of self-reported physician-diagnosed pulmonary fibrosis (PF) according to socio-demographics and WTC dust exposure (*N* = 19,300).

Variable at Enrollment	No. of PF	Person-Years (PY)	Rate (No. of PF/100,000 PY)	95% Confidence Interval
Total	73	199,113.6	36.7	29.2, 46.1
Age, year				
<45 (22–44)	29	107,890.0	26.9	18.7, 38.7
45–64	40	85,721.3	46.7	34.2, 63.6
≥65	4	5502.3	72.7	27.3, 193.6
Gender				
Male	61	154,690.0	39.4	30.7, 50.7
Female	12	44,427.5	27.0	15.3, 47.6
Race/Ethnicity				
Non-Latino white	45	151,627.3	29.7	22.2, 40.0
Non-Latino black	2	13,788.0	14.5	3.6, 58.0
Latino	17	22,846.3	74.4	46.3, 119.7
Asian	5	4334.8	115.3	48.0, 277.0
Other/unknown	4	6517.1	61.4	23.0, 163.5
Household income ($)				
<50,000	22	43,168.1	51.0	33.6, 77.4
≥50,000	46	140,651.4	32.7	24.5, 43.7
Missing	5	15,294.1	32.7	13.6, 78.5
Smoking status				
Never	31	112,062.1	27.7	19.5, 39.3
Former	19	55,556.1	34.2	21.8, 53.6
Current	22	30,295.1	72.6	47.8, 110.3
Missing	1	1200.3	83.3	11.8, 591.0
WTC dust/debris exposure level				
Quantile 1 (Low)	8	49,491.8	16.2	8.1, 32.3
Quantile 2 (Medium)	19	48,128.3	39.5	25.2, 61.9
Quantile 3 (High)	18	49,446.7	36.4	22.9, 57.8
Quantile 4 (Very high)	28	52,046.8	53.8	37.2, 77.9

**Table 3:** Adjusted hazard ratios (AHR) for self-reported physician-diagnosed pulmonary fibrosis (PF) according to socio-demographics and WTC dust exposure (*N* = 19,300).

Variable	AHR *	95% CI
Age at enrollment, year	1.05	1.03–1.08
Gender		
Female	referent	
Male	1.27	0.66–2.43
Race/Ethnicity		
Non-Latino white	referent	
Non-Latino black	0.47	0.11–1.95
Latino	2.45	1.37–4.37
Asian	4.62	1.81–11.79
Other/unknown	1.80	0.60–5.38
Household income at enrollment ($)		
<50,000	1.78	1.03–3.06
≥50,000	referent	
Missing	0.95	0.36–2.58
WTC dust/debris exposure level		
Quantile 1 (Low)	referent	
Quantile 2 (Medium)	2.51	1.09–5.80
Quantile 3 (High)	2.34	0.99–5.51
Quantile 4 (Very high)	4.51	1.96–10.38
P_trend_ for WTC exposure **	0.0043	

* Adjusted for demographic variables. ** Test for dose-response relationship.

**Table 4:** Distribution of comorbidity by self-reported physician-diagnosed pulmonary fibrosis (PF) status *.

Conditions Reported in W3 or W4	PF		No. PF	
	No.	%	No.	%
Physical conditions				
Gastroesophageal reflux disease (GERD)	50	69.4	6069	31.7
Obstructive sleep apnea (OSA)	50	68.5	4820	25.2
Asthma	44	60.3	4378	22.9
Chronic obstructive pulmonary disease (COPD) or emphysema	33	45.8	1406	7.3
Diabetes	15	20.8	2429	12.7
Mental disorders				
Depression	39	54.9	4583	26.0
PTSD	34	48.6	3593	20.6
Anxiety	25	36.8	2829	16.3

* Not mutually exclusive. *p* < 0.001 for all comorbidity listed in the table between two groups, except for diabetes (*p* = 0.030).
